# Chloride equilibrium potential in salamander cones

**DOI:** 10.1186/1471-2202-5-53

**Published:** 2004-12-05

**Authors:** Wallace B Thoreson, Eric J Bryson

**Affiliations:** 1 Departments of Ophthalmology and Pharmacology, University of Nebraska Medical Center, Omaha, NE, USA

## Abstract

**Background:**

GABAergic inhibition and effects of intracellular chloride ions on calcium channel activity have been proposed to regulate neurotransmission from photoreceptors. To assess the impact of these and other chloride-dependent mechanisms on release from cones, the chloride equilibrium potential (E_Cl_) was determined in red-sensitive, large single cones from the tiger salamander retinal slice.

**Results:**

Whole cell recordings were done using gramicidin perforated patch techniques to maintain endogenous Cl^- ^levels. Membrane potentials were corrected for liquid junction potentials. Cone resting potentials were found to average -46 mV. To measure E_Cl_, we applied long depolarizing steps to activate the calcium-activated chloride current (I_Cl(Ca)_) and then determined the reversal potential for the current component that was inhibited by the Cl^- ^channel blocker, niflumic acid. With this method, E_Cl _was found to average -46 mV. In a complementary approach, we used a Cl-sensitive dye, MEQ, to measure the Cl^- ^flux produced by depolarization with elevated concentrations of K^+^. The membrane potentials produced by the various high K^+ ^solutions were measured in separate current clamp experiments. Consistent with electrophysiological experiments, MEQ fluorescence measurements indicated that E_Cl _was below -36 mV.

**Conclusions:**

The results of this study indicate that E_Cl _is close to the dark resting potential. This will minimize the impact of chloride-dependent presynaptic mechanisms in cone terminals involving GABA_a _receptors, glutamate transporters and I_Cl(Ca)_.

## Background

Regulation of intracellular chloride levels results in a chloride equilibrium potential (E_Cl_) that is hyperpolarized with respect to the resting potential in many nerve cells, but depolarized in others [1-5]. For example, E_Cl _in salamander rod photoreceptors is 25 mV more positive than the dark resting potential [6]. The resting potential of cone photoreceptors in darkness is around -42 to -47 mV and estimates of E_Cl _in cones have ranged from -65 mV to -36 mV [7-11]. Cone photoreceptors possess a number of Cl^- ^conductances that help to shape their responses and synaptic output. As discussed below, the value of E_Cl _in cones is an important parameter for determining the strength and polarity of these effects.

It has been suggested GABA_a _receptors in the terminals of cones may mediate inhibitory synaptic feedback from horizontal cells to cones [8]. Under this hypothesis, the light-evoked hyperpolarization of horizontal cells causes a cessation of GABA release and this disinhibition leads to a "feedback depolarization" in cones. There is evidence both for [e.g., [8]] and against [e.g., [12,13]; see review in ref. [14]]) this hypothesis. However, one prediction of the hypothesis is that the Cl^- ^equilibrium potential (E_Cl_) must be negative to the resting potential in order for GABA disinhibition to depolarize a cone.

Cones possess prominent Ca^2+^-activated Cl^- ^currents (I_Cl(Ca)_) [15-17] activated by the influx of Ca^2+ ^through voltage-gated Ca^2+ ^channels as well as by release of Ca^2+ ^from intracellular stores [16]. Cl^- ^flux through I_Cl(Ca) _can be substantial: during a 1.4 sec depolarizing step, the charge movement accompanying activation of I_Cl(Ca) _is estimated to be 8.5 times that produced by activation of I_Ca _alone [16]. These large membrane currents can strongly influence photoreceptor responses, but the nature of these effects depends on the value of E_Cl_. If E_Cl _is positive to the resting potential, activation of I_Cl(Ca) _can boost depolarizing feedback responses from horizontal cells onto cones and produce prolonged, regenerative depolarizing responses lasting many seconds [9,18,19]. On the other hand, if E_Cl _is negative to the resting potential, activation of I_Cl(Ca) _can operate as a negative feedback mechanism to limit regenerative activation of Ca^2+ ^channels [15,17]. In addition to altering membrane potential, depletion of intracellular Cl^- ^can directly inhibit the open channel probability of single Ca^2+ ^channels, presumably by modifying an anion binding site on the intracellular surface of the channel [11]. In rods, where E_Cl _is positive to the resting potential, there is evidence for a negative feedback pathway between I_Ca _and I_Cl(Ca) _in which activation of I_Ca _stimulates I_Cl(Ca) _leading to a Cl^- ^efflux that in turn inhibits Ca^2+ ^channel activation [6,20]. If, however, E_Cl _in cones is negative to the membrane potential, then activation of I_Cl(Ca) _would stimulate an influx of Cl^- ^that would be expected to enhance Ca^2+ ^channel open probability [11].

Cone photoreceptors have presynaptic glutamate transporters that are coupled to Cl^- ^channels [21-23]. The transporters in cones have been shown to respond to glutamate released from their own terminals [24]. Whether synaptically released glutamate causes cones to hyperpolarize or depolarize depends on E_Cl_. Furthermore, analogous to the negative feedback from I_Cl(Ca) _onto I_Ca _described above, the chloride current produced by activation of glutamate transporters in rods can cause a Cl^- ^efflux that inhibits I_Ca _[25]. As with the feedback between I_Cl(Ca) _and I_Ca_, the strength and polarity of this potential interaction in cones depends on E_Cl_.

Given the importance of E_Cl _in determining the impact of various feedback mechanisms in the photoreceptor terminal, we determined E_Cl _in cone photoreceptors of the salamander retina using a combination of imaging with a chloride-sensitive dye and electrophysiological approaches.

## Results

In control superfusate, dark resting potentials of cones from slices prepared under visible light averaged -46.0 ± 2.00 mV (n = 9) after correcting for the liquid junction potential. This is nearly identical to the dark resting potentials of salamander cones prepared under infrared illumination (-46.8 ± 2.03 mV, n = 18).

**Figure 1:**
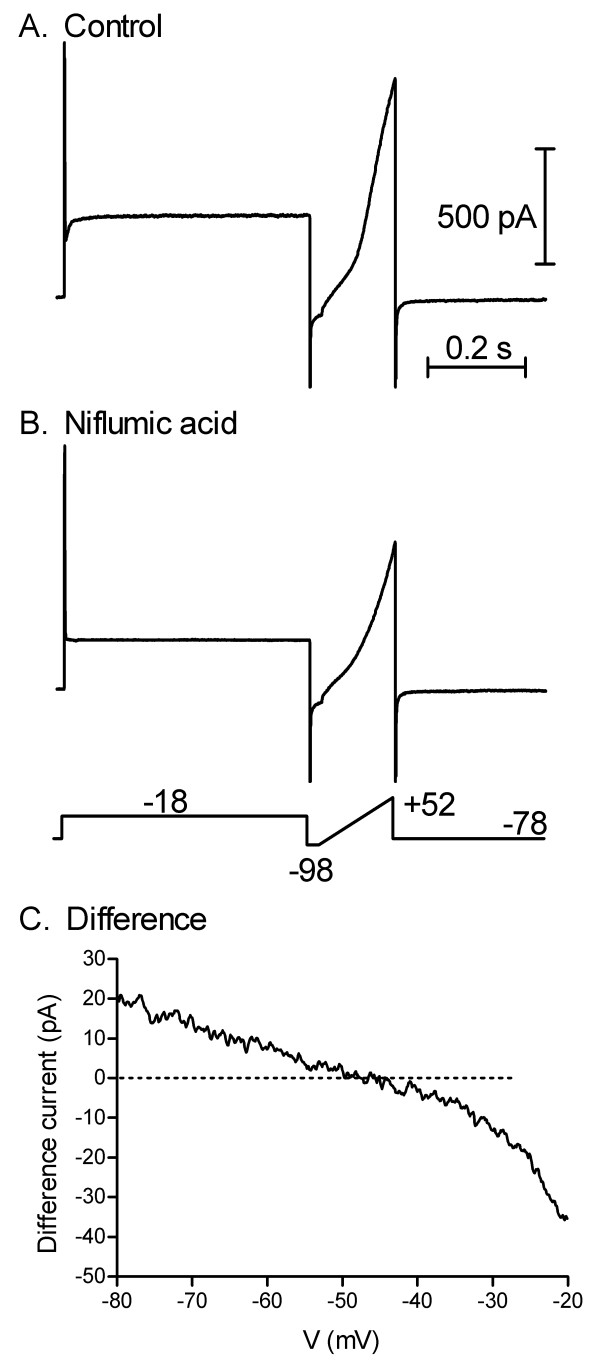
Cone E_Cl _estimated from the reversal of I_Cl(Ca)_. A) I_Cl(Ca) _tail current was activated by applying a 500 ms step from -78 to -18 mV during a gramicidin perforated patch whole cell recording from a rod. A ramp voltage protocol (-98 to +52 mV, 1 mV/ms) was applied during the tail current and begun 25 ms after termination of the step. B. The same protocol was then repeated in the presence of niflumic acid (0.1 mM) to inhibit I_Cl(Ca)_. C. The ramp current/voltage relationship obtained in control medium (A) was subtracted from that obtained in the presence of niflumic acid (B) to yield a niflumic acid-sensitive difference current that reversed in this cell around -46 mV (after correcting for a liquid junction potential of -8 mV).

To measure E_Cl_, I_Cl(Ca) _was recorded using gramicidin perforated patch whole cell recordings and activated by applying a 500 ms step from -78 to -8 mV. This depolarizing step typically evoked a sustained inward tail current arising largely from activation of I_Cl(Ca) _[19]. Only cells that exhibited an inward tail current were used for analysis. As shown in the example of Fig. 1A, the current/voltage relationship of a cone cell was assessed during the tail current by using a ramp voltage protocol (1 mV/ms from -98 to +52 mV) begun 25 ms after the end of the depolarizing step. The same protocol was then repeated after applying niflumic acid (0.1 mM; Fig. 1B). At this concentration, niflumic acid is a selective inhibitor of I_Cl(Ca) _in cones [[19]; niflumic acid may not be as selective in rods: [20,26]]. Subtracting the control ramp-evoked current from that obtained in the presence of niflumic acid yields the current/voltage profile for I_Cl(Ca) _(Fig. 1C). In the example shown in Fig. 1C, the difference current reversed around -46 mV. The reversal potential of the niflumic acid-sensitive difference current determined from 8 cones averaged -45.5 ± 2.5 mV. As a control for the possible perturbation of intracellular Cl^- ^by possible patch rupture, we repeated the same experiment using a pipette solution with only 3.5 mM Cl^-^. E_Cl _was not significantly different when measured using the low Cl^- ^pipette solution (-50.4 mV ± 3.4 mV; n = 7; p = 0.49, unpaired t-test). If patch rupture had occurred, E_Cl _would be expected to attain -89 mV with the low Cl^- ^pipette solution and -20 mV with the original pipette solution.

Bath application of GABA evoked small reversible inward currents that averaged -3.4 ± 0.3 pA at the holding potential of -78 mV (not shown). The small size of these currents may be due to receptor desensitization [27]. Consistent with results obtained from measurements of I_Cl(Ca)_, difference currents calculated from ramps applied before and during GABA application indicate that the GABA-evoked current reversed at -46.4 ± 2.7 mV (n = 9).

**Figure 2:**
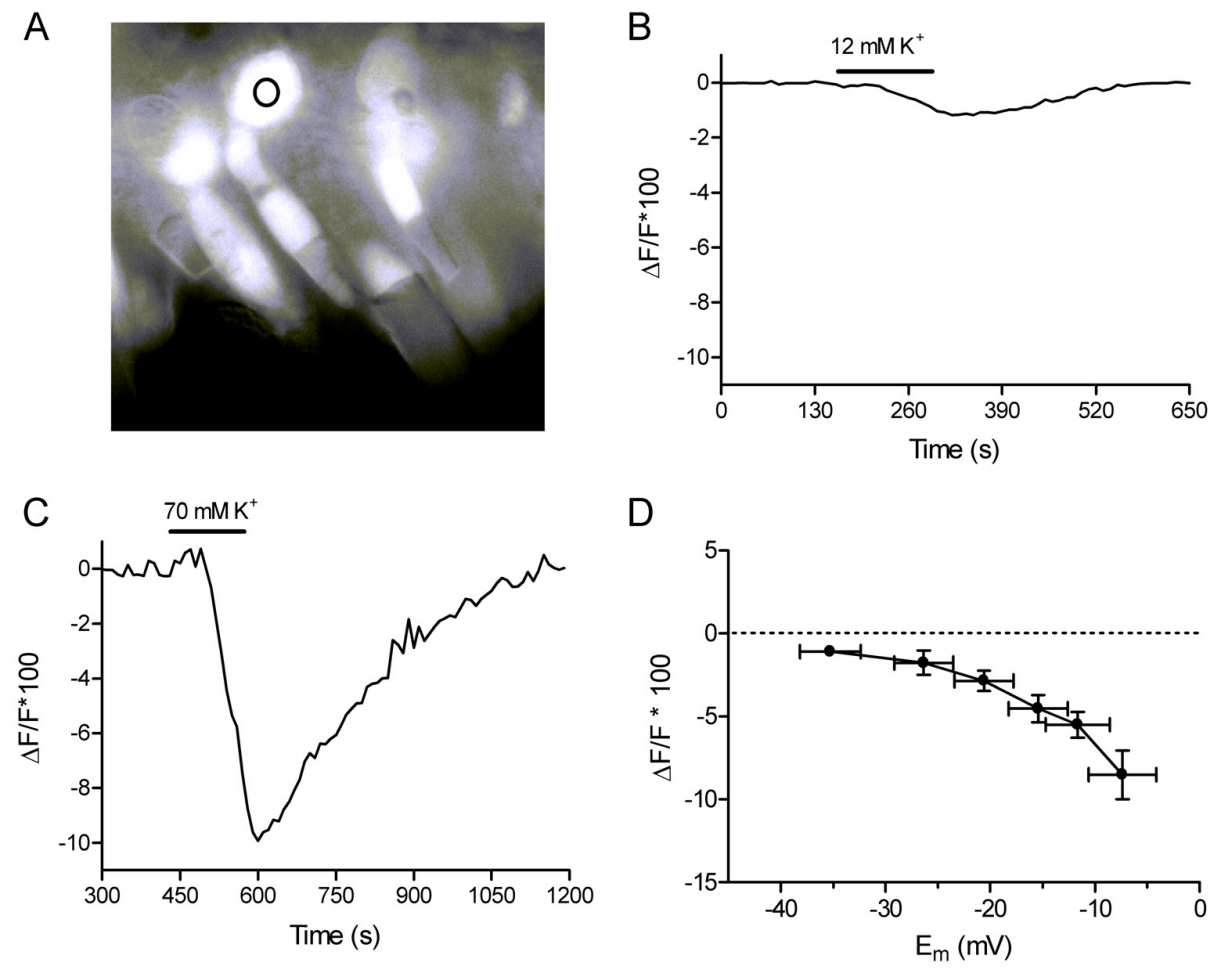
Intracellular chloride measurements. A. Example of a retinal slice fluorescently stained with the Cl^- ^sensitive dye MEQ. The circle indicates the somatic region of a cone in which MEQ measurements were made. B. An example of the reduction in MEQ fluorescence, indicating an increase in intracellular [Cl^-^], produced by application of 12.1 mM K^+ ^to a cone. C. A greater decrease in MEQ fluorescence was produced by application of 69.9 mM K^+^. Different cone from panel B. D. MEQ fluorescence changes produced by bath application of superfusate containing 12.1, 21.6, 31.2, 40.7, 50.3, or 69.9 mM [K^+^]. After correcting for liquid junction potentials, these high K^+ ^solutions depolarized cones to -35.3 ± 2.90, -26.3 ± 2.82, -20.6 ± 2.82, -15.4 ± 2.84, -11.6 ± 3.05, and -7.4 ± 3.25 mV (n = 9), respectively, determined in current clamp recordings using gramicidin-perforated patch recording techniques. The change in MEQ fluorescence relative to basal fluorescence (ΔF/F*100) is plotted against the cone membrane potential determined with each high K^+ ^solution.

In a complementary approach for measuring E_Cl_, we used a Cl-sensitive dye, MEQ, to examine the Cl^- ^flux that accompanied cone depolarization evoked by bath application of various high K^+ ^solutions (12, 22, 31, 41, 50 and 70 mM K^+^). In a separate set of experiments, we used gramicidin-perforated patch recording methods to measure the membrane potentials produced in cones by application of the different high K^+ ^solutions. Slices used for MEQ experiments and for measurement of membrane potentials in different solutions were prepared using similar techniques under visible illumination; control experiments showed that the fluorescent illumination used during MEQ experiments did not produce any further changes in the cone resting membrane potential (n = 3). An example of a retinal slice loaded with MEQ is shown in Fig. 2A. Measurements of MEQ fluorescence were made from the cone soma (circle, Fig. 2A). For a single wavelength dye such as MEQ, the change in fluorescence relative to basal fluorescence (ΔF/F) can be used as a measure of the change in ion concentration [28]. In the cone in Fig. 2B, bath application of 12 mM K^+^, which depolarized cones to -36 mV, produced a 1.2% decrease in MEQ fluorescence. Since MEQ fluorescence is quenched by Cl^- ^ions this indicates that depolarization to -36 mV stimulated an influx of Cl^- ^ions. Application of a solution with 70 mM K^+^, which depolarizes cones to -7 mV, produced a greater influx of Cl^- ^as evidenced by the 10% decrease in MEQ fluorescence seen in a different cone (Fig. 2C). Fig. 2D shows the average change in ΔF/F (x100) plotted as a function of the membrane potential evoked by the different high K^+ ^solutions. The finding that 12 mM K^+ ^consistently stimulated an influx of Cl^- ^indicates that the reversal potential must be below -36 mV.

## Discussion

The main finding of this study is that E_Cl _in salamander cones is close to the dark resting potential (~-46 mV). E_Cl _was found to be -46 mV from block of I_Cl(Ca) _by niflumic acid; small GABA-evoked currents reversed around the same potential. MEQ fluorescence changes produced by depolarization support these electrophysiological measurements by indicating that E_Cl _is below -36 mV.

There can be local variations of E_Cl _within cells [4]. Large single cones in the salamander retina do not have a distinct axon and terminal; synaptic proteins are instead located at the base of the soma [29]. MEQ measurements were made in the cell soma from a region adjacent to the synaptic ending (see Fig. 2). I_Cl(Ca) _is localized to the terminal region in rods (30) and these channels are probably also localized to the terminals of cones. Thus, the measurements in the present study are likely to provide estimates of E_Cl _in the synaptic terminal and adjacent regions of the cone cell. Measurements of intracellular Cl^- ^levels suggest that E_Cl _in the inner segment is not significantly different from that measured in the soma [11].

The finding that the Cl^- ^equilibrium potential is close to the resting potential does not necessarily mean that Cl^- ^is passively distributed. Electrophysiological experiments required that cells be voltage clamped at -70 mV for many minutes. Nonetheless, the value of E_Cl _determined from these electrophysiological experiments in which cells were voltage clamped at -70 mV was similar to the value estimated from MEQ studies in which cells were not voltage clamped and thus at their resting membrane potential. Results from experiments on the prolonged depolarization in cones also suggest that E_Cl _can be maintained indefinitely at a value above the membrane potential. The plateau phase of the prolonged depolarization, which largely reflects I_Cl(Ca) _activation [9,19], could remain above the membrane potential established by an adapting background for hours [9]. The ability of cones to maintain E_Cl _above the membrane potential may arise from activity of the Na/KCl cotransporter as shown in rods (20) as well as from other mechanisms (e.g., CLC-2) [2,31].

### Comparisons with other studies

E_Cl _in cones has been estimated in a number of previous studies. The most positive value for E_Cl _of -36 mV comes from calibration of MEQ fluorescence levels to determine the resting intracellular Cl^- ^concentrations in cones isolated from the salamander retina (11). However, these measurements showed a large variability (range of S.E.M.: -26.5 to -46.6 mV). The most negative estimate of E_Cl _comes from a study by Attwell et al [7] showing that the sign-reversing pathway from rods to cones reversed around -65 mV. Based on the presumption that this pathway involved disinhibition of GABAergic inputs into cones, this study has been interpreted as suggesting that E_Cl _is around -65 mV. However, more recent evidence questions whether the horizontal cell to cone feedback pathway thought to underlie this sign-reversing pathway from rods to cones is truly GABAergic [9,13,14]. Other studies have arrived at values for E_Cl _similar to those found in the present study. 1) By examining the polarity of GABA-evoked currents after patch rupture with either 12 or 24 mM Cl^- ^in the recording pipette, Kaneko and Tachibana [8] estimated E_Cl _to be around -47 mV in isolated turtle cones. 2) Based on the membrane potential attained by the plateau phase of the prolonged depolarization in turtle cones from the eyecup slice preparation, E_Cl _was estimated to be at or slightly above the dark resting potential of -42 mV [after correction for a liquid junction potential of -2 mV; ref. [9]]. 3) In a single recording from a salamander cone obtained with a Cl-sensitive electrode, Miller and Dacheux [32] found that E_Cl _was 2 mV more positive than the dark resting potential. 4) A slightly more negative value for E_Cl _was found in ruptured patch recordings from goldfish cones by examining the voltage dependence of the I_Cl(Ca) _tail current [10]. By extrapolating measurements back to the time of patch rupture, Kraaij et al [10] concluded that E_Cl _was ~-55 mV.

### Functional implications

I_Ca _in cones, like that of rods, can be inhibited by lowering extracellular Cl^- ^[33]. The inhibition of I_Ca _produced by lowering extracellular Cl^- ^appears to result from a reduction in intracellular Cl^- ^which in turn causes a reduction in the open probability of single Ca^2+ ^channels [11]. In rods, where E_Cl _is positive to the resting potential, activation of Cl^- ^channels leads to a Cl^- ^efflux thereby producing an inhibition of Ca^2+ ^channels [6,11,20]. The present results indicate that activation of Cl^- ^channels when the cell is at its resting potential would produce minimal changes in intracellular Cl^- ^in cones. Therefore, the feedback between I_Ca _and I_Cl(Ca) _postulated for rod photoreceptors [6,20] would be expected to be minimal in cones in darkness.

Another implication of the finding that E_Cl _is close to the dark resting potential is that the stimulation of Cl^- ^channels associated with glutamate transporters by glutamate released from cone terminals [24] would tend to stabilize the cell membrane potential near the dark potential. In rods, the Cl^- ^efflux accompanying activation of glutamate transporters appears to contribute to a glutamate-mediated inhibition of I_Ca _[25]. As with the feedback between I_Ca _and I_Cl(Ca) _considered in the previous paragraph, the finding that E_Cl _is near the resting potential leads to the prediction that in darkness there would be no Cl^- ^efflux accompanying glutamate transporter activation and therefore glutamate would not be expected to inhibit I_Ca_.

Cones hyperpolarize to light, although with prolonged illumination the membrane potential recovers to near the dark resting potential. The impact of chloride-dependent negative feedback between I_Cl(Ca) _and I_Ca _or the glutamate transporter chloride current and I_Ca _would be expected to increase as a cone hyperpolarizes in response to light. By reducing glutamate release, these chloride-dependent negative feedback mechanisms might thus contribute to making post-synaptic responses more transient.

The finding that E_Cl _is near the resting potential of cones indicates that GABAergic disinhibition near the dark potential should produce little membrane potential change. This result is inconsistent with the postulated role for GABA in generating the feedback depolarization [8] and supports other studies suggesting that GABA is not directly responsible for horizontal to cone feedback [9,13,14].

## Conclusions

Electrophysiological measurements, supported by experiments using chloride-sensitive dyes, indicate that E_Cl _in salamander cones is close to the dark resting membrane potential. By minimizing the trans-membrane flux of chloride, this will minimize the presynaptic impact of GABA_a _receptors, I_Cl(Ca)_, and glutamate transporter chloride channels.

## Methods

### Tissue preparation

E_Cl _is positive to the resting potential of many neurons in the immature brain [5]. Based on their size, the neotenous tiger salamanders (*Ambystoma tigrinum*, 15–25 cm) used in these experiments are thought to be 2–7 years old out of a life span of ~12 years (34).

Salamanders were handled humanely in accordance with protocols approved by the Institutional Animal Care and Use Committee at the University of Nebraska Medical Center. Chilled salamanders were rapidly decapitated, an eye was enucleated, and the front of the eye was removed. The resulting eyecup was cut into three or four pieces and a single piece was placed vitreal surface down onto a piece of filter paper (2 × 5 mm, Millipore type AAWP, 0.8 μm pores). After adhering to the filter paper, the retina was isolated under chilled amphibian superfusate and cut into 125 μm slices using a razor blade tissue chopper (Stoelting Co., Wood Dale, IL). The slices were rotated 90° to view the retinal layers when placed under a water immersion objective (60X, 1.0 NA) on an upright fixed stage microscope (EF 600, Nikon Inc., USA). Slices were prepared under visible light but recordings were performed in darkness. All experiments were done using red-sensitive large single cones selected by anatomical criteria [35].

### Solutions and perfusion

Solutions were applied with a single-pass, gravity-feed perfusion system (1 ml/min). The normal amphibian superfusate contained (in mM): 111 NaCl, 2.5 KCl, 1.8 CaCl_2_, 0.5 MgCl_2_, 10 *N*-2-hydroxyethylpiperazine-*N*' 2-ethanesulfonic acid (HEPES), and 5 glucose (pH 7.8). The osmolarity was measured with a vapor pressure osmometer (Wescor, Logan, UT) and adjusted, if necessary, to 242 ± 5 mOsm. For high K^+ ^solutions, various quantities of NaCl were replaced with equimolar KCl. Niflumic acid was diluted (1:10,000) from DMSO stock solutions. Unless otherwise specified, chemicals were obtained from Sigma/Aldrich/RBI (St. Louis, MO).

### Electrophysiology

Patch pipettes were pulled on a PP-830 vertical puller (Narishige USA, New York) from borosilicate glass pipettes (1.2 mm O.D., 0.95 mm I.D., with internal filament) and had tips of ~1 μm outer diameter with resistances of 10 to 15 MΩ. To maintain endogenous levels of intracellular Cl^-^, we obtained perforated patch whole cell recordings using the cation channel, gramicidin [36]. Gramicidin was dissolved in ethanol (5 mg/ml) and then added to the pipette electrolyte solution to achieve a final concentration of 5 μg/ml. For current clamp measurements of membrane potentials, the pipette electrolyte solution contained (in mM): 54 KCl, 61.5 KCH_3_SO_4 _(Pfaltz and Bauer, Waterbury, CT), 3.5 NaCH_3_SO_4_, 10 HEPES. The pH was adjusted to 7.2 with KOH. The liquid junction potential (LJP) of this solution was estimated to be -7 mV using the junction potential calculator of PClamp (Axon Instruments). Membrane potential values reported throughout this manuscript were corrected for the LJP. For experiments with niflumic acid or GABA, pipettes were typically filled with a solution containing (in mM): 54 CsCl, 61.5 CsCH_3_SO_3_, 3.5 NaCH_3_SO_4_, 10 HEPES (LJP = -8 mV). In some experiments, a low Cl^- ^pipette solution was used containing: 115.5 mM CsCH_3_SO_3_, 3.5 NaCl, 10 HEPES (LJP = -10 mV). The pH of both solutions was adjusted to 7.2 with CsOH. The osmolarity of pipette solutions were also adjusted, if necessary, to 242 ± 5 mOsm. Recordings were made using an Axopatch 200B amplifier (Axon Instruments Inc., Union City, CA) and PClamp 8 software (Axon Instruments). Cell input resistance calculated using a step from -70 to -90 mV averaged 695 ± 111 MΩ. Access resistance estimated from the peak of the capacitative transient averaged 30.7 ± 4.8 MΩ (n = 24).

### Imaging experiments

Digital fluorescent images were obtained with a cooled CCD camera (SensiCam, Cooke Corp., Auburn Hills, MI). Axon Imaging Workbench (AIW 2.2, Axon Instruments Inc., Union City, CA) was used to control the camera, filter wheel, and image acquisition. Pixel binning (2 × 2) of the images was used to decrease acquisition time to ≤1 s. Images were acquired at 5 to 10 s intervals during experimental trials.

For measurements of [Cl^-^]_i _we used the dye, 6-methoxy-N-ethylquinolinium iodide (MEQ, Molecular Probes, Eugene, OR) [37]. MEQ was loaded into cells after reducing it to DiH-MEQ by adding 30 μM sodium borohydride (100 μl) to MEQ (5 mg) under a continuous stream of nitrogen gas [38]. DiHMEQ enters cells during the incubation period (15 min) where it is oxidized and retained in the form of MEQ. Fluorescence emission decreases as Cl^- ^quenches MEQ. The slow exponential decay in MEQ fluorescence due to dye leakage and bleaching was determined from a 3 min. series of control measurements prior to drug application and subtracted before analysis [11,20].

Variance is reported as ± S.E.M.

## Authors' contributions

WT conceived the study and drafted the manuscript. WT and EB participated in all aspects of the experiments but most recordings were performed by EB. Both authors have read and approved the manuscript.

## References

[B1] Alvarez-Leefmans FJ, Gamino SM, Giraldez F, Nogueron I (1988). Intracellular chloride regulation in amphibian dorsal root ganglion neurones studied with ion-selective microelectrodes. J Physiol.

[B2] Staley K, Smith R, Schaack J, Wilcox C, Jentsch TJ (1996). Alteration of GABA_A _receptor function following gene transfer of the CLC-2 chloride channel. Neuron.

[B3] Reuter D, Zierold K, Schroder WH, Frings S (1998). A depolarizing chloride current contributes to chemoelectrical transduction in olfactory sensory neurons in situ. J Neurosci.

[B4] Vardi N, Zhang LL, Payne JA, Sterling P (2000). Evidence that different cation chloride cotransporters in retinal neurons allow opposite responses to GABA. J Neurosci.

[B5] Ben-Ari Y (2002). Excitatory actions of gaba during development: the nature of the nurture. Nature Rev Neurosci.

[B6] Thoreson WB, Stella SL, Bryson EJ, Clements J, Witkovsky P (2002). D2-like dopamine receptors promote interactions between calcium and chloride channels that diminish rod synaptic transfer in the salamander retina. Vis Neurosci.

[B7] Attwell D, Werblin FS, Wilson M, Wu SM (1983). A sign-reversing pathway from rods to double and single cones in the retina of the tiger salamander. J Physiol.

[B8] Kaneko A, Tachibana M (1986). Effects of gamma-aminobutyric acid on isolated cone photoreceptors of the turtle retina. J Physiol.

[B9] Thoreson WB, Burkhardt DA (1991). Ionic influences on the prolonged depolarization of turtle cones in situ. J Neurophysiol.

[B10] Kraaij DA, Spekreijse H, Kamermans M (2000). The nature of surround-induced depolarizing responses in goldfish cones. J Gen Physiol.

[B11] Thoreson WB, Nitzan R, Miller RF (2000). Chloride efflux inhibits single calcium channel open probability in vertebrate photoreceptors: chloride imaging and cell-attached patch-clamp recordings. Vis Neurosci.

[B12] Thoreson WB, Burkhardt DA (1990). Effects of synaptic blocking agents on the depolarizing responses of turtle cones evoked by surround illumination. Vis Neurosci.

[B13] Verweij J, Kamermans M, Spekreijse H (1996). Horizontal cells feed back to cones by shifting the cone calcium-current activation range. Vision Res.

[B14] Kamermans M, Spekreijse H (1999). The feedback pathway from horizontal cells to cones. A mini review with a look ahead. Vision Res.

[B15] Maricq AV, Korenbrot JI (1988). Calcium and calcium-dependent chloride currents generate action potentials in solitary cone photoreceptors. Neuron.

[B16] Barnes S, Hille B (1989). Ionic channels of the inner segment of tiger salamander cone photoreceptors. J Gen Physiol.

[B17] Yagi T, Macleish PR (1994). Ionic conductances of monkey solitary cone inner segments. J Neurophysiol.

[B18] Burkhardt DA, Gottesman J, Thoreson WB (1988). Prolonged depolarization in turtle cones evoked by current injection and stimulation of the receptive field surround. J Physiol.

[B19] Barnes S, Deschenes MC (1992). Contribution of Ca and Ca-activated Cl channels to regenerative depolarization and membrane bistability of cone photoreceptors. J Neurophysiol.

[B20] Thoreson WB, Bryson EJ, Rabl K (2003). Reciprocal interactions between calcium and chloride in rod photoreceptors. J Neurophysiol.

[B21] Eliasof S, Arriza JL, Leighton BH, Kavanaugh MP, Amara SG (1998). Excitatory amino acid transporters of the salamander retina: identification, localization, and function. J Neurosci.

[B22] Eliasof S, Werblin F (1993). Characterization of the glutamate transporter in retinal cones of the tiger salamander. J Neurosci.

[B23] Picaud SA, Larsson HP, Grant GB, Lecar H, Werblin F (1995). Glutamate-gated chloride channel with glutamate-transporter-like properties in cone photoreceptors of the tiger salamander. J Neurophysiol.

[B24] Picaud S, Larsson HP, Wellis DP, Lecar H, Werblin F (1995). Cone photoreceptors respond to their own glutamate release in the tiger salamander. Proc Natl Acad Sci USA.

[B25] Rábl K, Bryson EJ, Thoreson WB (2003). Activation of glutamate transporters in rods inhibits presynaptic calcium currents. Vis Neurosci.

[B26] Satoh TO, Yamada M (2001). Niflumic acid reduces the hyperpolarization-activated current (I(h)) in rod photoreceptor cells. Neurosci Res.

[B27] Epstein R, Grundfest H (1970). Desensitization of gamma aminobutyric acid (GABA) receptors in muscle fibers of the crab Cancer borealis. J Gen Physiol.

[B28] Helmchen F, Yuste R, Lanni F, Konnerth A (2000). Calibration of fluorescent calcium indicators. Imaging Neurons: A Laboratory Manual.

[B29] Sherry DM, Yang H, Standifer KM (2001). Vesicle-associated membrane protein isoforms in the tiger salamander retina. J Comp Neurol.

[B30] Macleish PR, Nurse CA (2000). Ion channel compartments in photoreceptors [abstract]. Invest Ophthalm Vis Sci.

[B31] Bösl MR, Stein V, Hubner C, Zdebik AA, Jordt SE, Mukhopadhyay AK, Davidoff MS, Holstein AF, Jentsch TJ (2001). Male germ cells and photoreceptors, both dependent on close cell-cell interactions, degenerate upon ClC-2 Cl^- ^channel disruption. EMBO J.

[B32] Miller RF, Dacheux RF (1983). Intracellular chloride in retinal neurons: measurement and meaning. Vision Res.

[B33] Thoreson WB, Nitzan R, Miller RF (1997). Reducing extracellular Cl^- ^suppresses dihydropyridine-sensitive Ca^2+ ^currents and synaptic transmission in amphibian photoreceptors. J Neurophysiol.

[B34] Townes-Anderson E, Colantonio A, St Jules RS (1998). Age-related changes in the tiger salamander retina. Exp Eye Res.

[B35] Stella SL, Thoreson WB (2000). Differential modulation of rod and cone calcium currents in tiger salamander retina by D2 dopamine receptors and cAMP. Eur J Neurosci.

[B36] Kyrozis A, Reichling DB (1995). Perforated-patch recording with gramicidin avoids artifactual changes in intracellular chloride concentration. J Neurosci Methods.

[B37] Biwersi J, Verkman AS (1991). Cell-permeable fluorescent indicator for cytosolic chloride. Biochemistry.

[B38] Woll E, Gschwentner M, Furst J, Hofer S, Buemberger G, Jungwirth A, Frick J, Deetjen P, Paulmichl M (1996). Fluorescence-optical measurements of chloride movements in cells using the membrane-permeable dye diH-MEQ. Pflugers Arch.

